# Beyond SCORE2: Rethinking Cardiovascular Risk Assessment in a Very-High-Risk European Setting—A Narrative Review and Proposal of the ROMA-CV Algorithm for Romania

**DOI:** 10.3390/jcm15145490

**Published:** 2026-07-13

**Authors:** Daniel Miron Brie, Cristian Mornoș, Roxana Popescu, Alina Diduța Brie

**Affiliations:** 1 Cardiovascular Disease Institute Timisoara, Gheorghe Adam Street, No. 13A, 300310 Timisoara, Romania; mornos.cristian@umft.ro; 2 Research Center of the Institute of Cardiovascular Diseases, Gheorghe Adam Street, No. 13A, 300310 Timisoara, Romania; 3 Department of Cardiology, “Victor Babeș” University of Medicine and Pharmacy Timisoara, Eftimie Murgu Square, No. 2, 300041 Timisoara, Romania; 4 Department of Cell and Molecular Biology, “Victor Babeș” University of Medicine and Pharmacy, Tudor Vladimirescu Street, No. 14, 300174 Timisoara, Romania; popescu.roxana@umft.ro (R.P.); alina.brie@umft.ro (A.D.B.); 5 ANAPATMOL Research Center, “Victor Babeș” University of Medicine and Pharmacy, Tudor Vladimirescu Street, No. 14, 300174 Timisoara, Romania; 6 “Louis Țurcanu” Emergency Children Hospital, Doctor Iosif Nemoianu Street, No. 2, 300011 Timisoara, Romania

**Keywords:** cardiovascular risk assessment, SCORE2, Pooled Cohort Equations, primary prevention, ESC guidelines, ACC/AHA guidelines, lipoprotein(a), coronary artery calcium, subclinical atherosclerosis, Romania

## Abstract

**Background:** Cardiovascular disease (CVD) remains the leading cause of mortality across the European Union (EU), with a fourfold to fivefold east–west gradient in standardized circulatory-disease mortality. Romania ranks second highest in the EU (787 per 100,000 inhabitants in 2023). Primary-prevention practice is organized around two quantitative frameworks—the 2021 European Society of Cardiology (ESC) SCORE2/SCORE2-OP system and the 2018/2019 American College of Cardiology/American Heart Association (ACC/AHA) Pooled Cohort Equations (PCE)—which converge on therapy but diverge on patient selection. **Methods:** We conducted a structured narrative review (SANRA-compliant) of contemporary primary-prevention guidelines, validation studies, key trials of risk-modifier interventions, and Romanian epidemiological data through April 2026. On this basis, we developed ROMA-CV (Risk Of Multifactorial Atherosclerosis—CardioVascular) as a conceptual, country-specific risk-stratification framework anchored to existing Class I/IIa recommendations or Level A/B evidence, rather than as a fully developed, ready-to-use clinical tool **Results:** Four structural limitations of SCORE2 are clinically consequential in Romania: (i) an age floor of 40 years that excludes the population in which premature myocardial infarction is most preventable; (ii) a country-level calibration coefficient applied to individuals; (iii) permissive treatment of lipoprotein(a) [Lp(a)]; and (iv) an under-emphasis of subclinical-atherosclerosis imaging. We propose ROMA-CV (Risk Of Multifactorial Atherosclerosis—Cardiovascular), a four-step algorithm retaining SCORE2 as the quantitative spine while embedding once-in-a-lifetime Lp(a) measurement, a mandatory amplifier checklist, and selective coronary artery calcium (CAC) or carotid/femoral ultrasound imaging. **Conclusions:** ROMA-CV is a hypothesis-generating proposal that operationalizes existing evidence-based recommendations into a deterministic Romanian pathway aligned with the 2025–2030 Romanian National Plan for Non-Communicable Diseases and the EU Cardiovascular Health Plan. The algorithm is not a validated clinical decision tool and requires prospective external validation in Romanian cohorts—alongside feasibility, cost-effectiveness, and implementation studies—before any consideration of routine clinical adoption.

## 1. Introduction

Cardiovascular disease (CVD) remains the largest single contributor to mortality in the European Union (EU). In 2023, circulatory diseases caused approximately one-third of all deaths and was the leading cause in 21 of the 27 Member States [1]. The standardized death rate from circulatory disease across the EU was 313 per 100,000 inhabitants, but this aggregate figure obscures an east–west gradient that is unparalleled in non-communicable-disease epidemiology: Bulgaria 923, Romania 787, and Latvia 726 per 100,000, versus France 163, Spain 200, and Denmark 208—a fourfold to fivefold ratio between citizens of the same single market sharing identical pharmacological options [1]. Romania ranks second highest in the Union, and the gap is widening rather than closing [1,2].

The biological levers of this excess have been mapped repeatedly. The INTERHEART case–control study across 52 countries demonstrated that nine readily measurable, mostly modifiable risk factors account for more than 90% of the population-attributable risk of a first myocardial infarction (MI), with remarkable consistency across regions, sexes, and ethnic groups [3]. The PURE study confirmed in a contemporary 21-country prospective cohort that the same risk-factor cluster explains roughly 70% of incident CVD, with the largest residual burden in low- and middle-income countries [4]. The corollary is therapeutically optimistic: most premature CVD events are theoretically preventable with tools already in the formulary.

Translating that potential into population benefit depends on a single decision: who is offered preventive therapy, and on what evidence. Quantitative risk estimation is the gating step of every contemporary primary-prevention guideline, determining whether a patient receives a statin, an antihypertensive, a sodium-glucose co-transporter 2 (SGLT2) inhibitor, or behavioral counseling alone. Two frameworks dominate global practice. The 2021 European Society of Cardiology (ESC) guideline on CVD prevention installed the SCORE2 and SCORE2-Older Persons (SCORE2-OP) algorithms as the standard short-term risk estimators for apparently healthy adults in Europe [5]. The 2018/2019 American College of Cardiology/American Heart Association (ACC/AHA) primary-prevention guideline operationalizes the Pooled Cohort Equations (PCE), with risk-enhancing factors and selective use of coronary artery calcium (CAC) scoring [6,7]. SCORE2/SCORE2-OP are explicitly calibrated and recommended for use in European populations, with country- or region-specific risk charts, whereas the ACC/AHA PCE were derived in U.S. cohorts and are intended primarily for North American practice. In day-to-day clinical care, these tools are therefore not truly interchangeable; European clinicians predominantly rely on SCORE2/SCORE2-OP, while PCE are used mainly in U.S. settings, although individual practitioners may consult both when managing patients with cross-border care or ambiguous risk profiles. This population- and context-specific use is central to the interpretability of the head-to-head comparison that follows and to the rationale for a Romanian country-specific framework.

The two frameworks share the same evidence base on therapy but diverge sharply on whom to treat, when to start, and how to handle uncertainty at the borderline.

This divergence is not merely academic. A recent analysis in The Lancet Global Health showed that approximately 40% of major CVD events occur in adults with no prior CVD who are not classified as high risk by current scores, indicating that the gating step itself is the rate-limiting failure of contemporary prevention [8]. For SCORE2 specifically, contemporary validation work has shown systematic underestimation in older European primary-care populations [9], and SCORE2’s age floor of 40 years categorically excludes the segment of the population in which premature MI is most common and most preventable [10,11]. Three additional structural issues compound the problem: (i) SCORE2’s country-level calibration coefficient is applied to individuals whose biological exposure is determined by their physiology rather than their postal code [5]; (ii) lipoprotein(a) [Lp(a)]—a globally prevalent, genetically determined causal risk factor—is mentioned but not embedded in the algorithm [12,13]; and (iii) subclinical atherosclerosis, the strongest mechanistic predictor available in primary prevention, is treated as optional rather than decisive [14,15].

Romania sits at the unfavorable end of every relevant axis. It carries one of the highest CVD mortality burdens in the EU [1]; it is classified as a “very-high-risk” SCORE2 region [5]; and approximately 3.15 million Romanian citizens—about 16% of the resident population—currently live in other EU Member States, where they are recalibrated to entirely different SCORE2 strata despite identical genetics, lifestyle, and disease trajectory [16]. The 2025–2030 Romanian National Plan for the Prevention and Control of Non-Communicable Diseases commits approximately €204 million and 10–12 regional prevention centers specifically to cardiovascular and metabolic risk reduction, creating an operational vehicle through which a more accurate national risk-stratification algorithm could be deployed at scale [17].

The aim of this article is threefold. First, to compare the ESC 2021 and ACC/AHA 2018/2019 primary-prevention frameworks head-to-head, identifying where structural choices converge or diverge clinically. Second, to dissect four structural shortcomings of the SCORE2-based ESC approach that we judge clinically consequential in Romanian practice. Third, to propose a country-specific, evidence-based primary-prevention algorithm—ROMA-CV (Risk OfMultifactorial Atherosclerosis—CardioVascular)—that integrates SCORE2 with universal once-in-a-lifetime Lp(a) measurement, a mandatory amplifier checklist, and selective CAC or carotid/femoral ultrasound imaging. Throughout, our objective is descriptive and constructive: we treat SCORE2 not as a tool to be replaced, but as a foundation that must be augmented where its assumptions are weakest. ROMA-CV is presented as a hypothesis-generating proposal that requires prospective external validation before clinical adoption. In ROMA-CV, SCORE2/SCORE2-OP are explicitly used as a quantitative “floor” rather than a definitive verdict; individual-level modifiers (Lp(a), family history, risk-enhancing conditions) and subclinical-atherosclerosis imaging can upgrade risk estimates when SCORE2 underestimates susceptibility. The age blind spot below 40 years is addressed by incorporating lifetime-risk concepts and targeted assessment of high-risk young adults, and the ecological limitations of the country calibration coefficient are mitigated by prioritizing individual biological and exposure profiles over residence alone when discordant. In this way, the proposed framework seeks to preserve the strengths of SCORE2—its large European derivation and ESC integration—while operationally buffering the specific limitations identified in very-high-risk settings such as Romania.

**Key Contributions**

- A head-to-head comparison of the 2021 ESC and 2018/2019 ACC/AHA frameworks identifies an upstream divergence in patient selection that is clinically consequential in very-high-risk settings.
- Four structural limitations of SCORE2 in Romania are characterized: age floor 40 years, country coefficient applied to individuals, permissive Lp(a) handling, and optional rather than gating role for CAC/carotid imaging.
- ROMA-CV is proposed as a four-step country-specific algorithm: universal baseline + once-lifetime Lp(a) → quantitative risk → mandatory amplifier checklist → selective CAC or carotid/femoral ultrasound.
- Implementation is aligned with the 2025–2030 Romanian National Plan and the EU Cardiovascular Health Plan; ROMA-CV is explicitly framed as a proposal that requires prospective validation.

## 2. Materials and Methods

### 2.1. Study Design

This article is a structured narrative review with a proposal component, conducted in accordance with the Scale for the Assessment of Narrative Review Articles (SANRA) and the methodological recommendations for narrative reviews of clinical guidelines [18,19]. To enhance methodological transparency and facilitate editorial verification, a SANRA adherence checklist for this narrative review is provided in Supplementary Table S1. Because the central deliverable is a clinical algorithm rather than a quantitative effect estimate, the review was not registered with PROSPERO and did not follow the PRISMA reporting standard for systematic reviews. The methodological rationale, search strategy, inclusion/exclusion logic, and synthesis steps are nonetheless made transparent and reproducible below.

### 2.2. Information Sources and Search Strategy

We searched MEDLINE (PubMed), Embase, and the Cochrane Library from inception to 30 April 2026, supplemented by manual searches of the websites of the European Society of Cardiology, American College of Cardiology, American Heart Association, European Atherosclerosis Society, National Lipid Association, the National Institute for Public Health of Romania, the European Commission, and the Romanian Ministry of Health. We also searched ClinicalTrials.gov for late-phase trials of agents targeting Lp(a) and inflammation, and the Eurostat and World Health Organization (WHO) databases for population statistics. Reference lists of all retrieved guidelines, consensus statements, and major reviews were screened for additional citations.

The core PubMed search combined four concept blocks: (i) primary cardiovascular prevention and risk estimation (e.g., “SCORE2”[tiab] OR “Pooled Cohort Equations”[tiab] OR “cardiovascular risk assessment”[Mesh]); (ii) guideline frameworks (e.g., “ESC”[tiab] OR “ACC/AHA”[tiab] OR “primary prevention guideline”[tiab]); (iii) risk modifiers (e.g., “lipoprotein(a)” OR “coronary artery calcium” OR “carotid plaque” OR “subclinical atherosclerosis”); and (iv) implementation context for Romania (e.g., “Romania”[tiab] OR “Eastern Europe”[tiab] OR “very high risk region”[tiab]).

### 2.3. Eligibility and Selection

We included: (i) the most recent full text of the ESC, ACC/AHA, EAS and NLA primary-prevention and lipid-management guidelines; (ii) the SCORE2, SCORE2-OP, SCORE2-Diabetes, and PCE derivation and external-validation papers; (iii) prospective cohort studies and randomized trials that informed contemporary risk-amplifier and imaging recommendations (PESA, MESA, BioImage, ARIC, SCOT-HEART, CONFIRM, ROBINSCA, EISNER); (iv) consensus statements on Lp(a), inflammation and lifetime risk; (v) the late-phase therapeutic trials of Lp(a) lowering (Lp(a)HORIZON, OCEAN(a)-Outcomes, ACCLAIM-Lp(a), KRAKEN); and (vi) national surveillance, mortality, and policy documents relevant to Romania. We excluded narrative reviews without primary data, conference abstracts not subsequently published, and articles not available in English or Romanian. When multiple publications addressed the same dataset, we cited the most recent comprehensive source together with the original derivation paper. A detailed reconstruction of the database and supplementary search strategy, together with prespecified inclusion and exclusion criteria and the staged study-selection workflow, is provided in Appendix A.

### 2.4. Synthesis and Algorithm Development

The comparative synthesis (Section 3, Section 4 and Section 5 and Table 1) followed a head-to-head framework: each clinically actionable decision node (eligibility, age window, score, intermediate categories, role of imaging, role of Lp(a), LDL-C targets, role of polypill/aspirin) was extracted independently for ESC 2021 and ACC/AHA 2018/2019, then reconciled in tabular form. Discrepancies between the guideline text and post-publication validation evidence were explicitly retained rather than smoothed over.

The proposed ROMA-CV algorithm (Section 7) was constructed through three iterative steps. First, we mapped the four identified shortcomings of SCORE2 (age floor, country coefficient, Lp(a), subclinical atherosclerosis) to evidence-based mitigations available in the published literature. Second, we required that every algorithm step rest on at least one Class I or Class IIa recommendation in either the ESC 2021 or ACC/AHA 2018/2019 framework, or on Level A/B evidence from a peer-reviewed primary source; no novel thresholds were created. Third, we constrained the proposal to interventions that are realistically deployable within Romania’s existing reimbursement and laboratory infrastructure, with explicit reference to the €204 million 2025–2030 National Plan [17].

### 2.5. Ethical Considerations

No primary data were generated, and no patient-level information was used; ethics committee approval was not required. Informed consent was not applicable. The study does not involve human or animal subjects. The reference list was organized with the assistance of an artificial intelligence tool (Claude Opus 4.8, Anthropic) to facilitate formatting and consistency. Generative artificial intelligence (Claude, Anthropic) was used in accordance with current recommendations on disclosure of AI-assisted technologies in scholarly work. The tool did not contribute to study conception, data collection, data analysis, interpretation of results, or drafting of the manuscript, and all references were independently checked by the authors for accuracy and completeness

## 3. The 2021 ESC Prevention Framework

In the following sections, we explicitly separate guideline-endorsed recommendations from emerging evidence. Framework statements refer to the original ESC 2021 and ACC/AHA 2018/2019 guideline text (including their cited classes and levels of recommendation), whereas emerging evidence refers to post-guideline validation studies, cohort data, and our interpretation of their implications. This distinction is maintained throughout the discussion of SCORE2, SCORE2-OP, PCE, and PREVENT, so that readers can more clearly appraise the strength and endorsement level of each statement. The following description reflects the Class I/II recommendations of the 2021 ESC Guidelines on CVD prevention in clinical practice, which installed SCORE2/SCORE2-OP as the standard primary-prevention risk estimators in Europe.

The 2021 ESC Guidelines on CVD prevention in clinical practice introduced the most substantial overhaul of European primary-prevention practice in 15 years [5]. Its central innovations are (i) replacement of the original SCORE chart with the SCORE2 and SCORE2-OP algorithms, (ii) explicit recalibration to four geographically defined population risk regions, (iii) age-dependent thresholds for high and very-high risk, and (iv) a stepwise treatment-intensification logic that allows risk-modifier upgrading.

### 3.1. SCORE2 and SCORE2-OP: Derivation and Structure

Framework. The 2021 ESC guideline positions SCORE2 and SCORE2-OP as the standard quantitative risk-estimation tools for apparently healthy European adults, with SCORE2 applied in adults aged 40–69 years and SCORE2-OP in those aged 70–89 years; both estimate 10-year first fatal and non-fatal cardiovascular events and are intended to be interpreted within the four ESC regional calibration strata.

Emerging evidence. SCORE2 was derived from individual-participant data on 677,684 participants pooled from 44 European cohorts, with external validation in 1,133,181 participants from 25 cohorts in 15 additional countries [20]. It estimates the 10-year risk of fatal and non-fatal cardiovascular events (MI or stroke)—a deliberate departure from the original SCORE, which estimated fatal events only. Inputs are age (40–69 years), sex, smoking status, systolic blood pressure, and non-HDL cholesterol. SCORE2-OP, derived from 28,503 individuals aged ≥ 65 years in the Cohort of Norway and validated in CONOR, ESTHER, KORA, Tromsø, and other registries, extends the model to ages 70–89 [21]. SCORE2-Diabetes, published in 2023, extends the family to adults with type 2 diabetes by incorporating age at diabetes diagnosis, HbA1c, and estimated glomerular filtration rate [22].

### 3.2. The Four-Region Calibration

Framework. Because cardiovascular event rates differ severalfold across Europe, SCORE2 is calibrated to four risk regions (low, moderate, high, very high) defined by national age-standardized cardiovascular mortality [5,20]. Romania is classified as a very-high-risk country, alongside Bulgaria, Latvia, Lithuania, Hungary, and Slovakia, among others. The clinical effect is substantial: identical biological inputs yield 10-year predicted risks that differ by approximately a factor of 3.5–4.0 between the lowest and highest regions. The political logic—calibration to local mortality—is empirically necessary. The clinical logic—that an individual’s risk reflects his or her country of residence rather than physiology—is precisely where the framework becomes vulnerable and is the principal subject of Section 6.

Emerging evidence. The region-based recalibration is guideline endorsed, but its application at individual level raises post-guideline concerns regarding ecological inference, calibration drift, and within-country heterogeneity, issues discussed in detail in Section 6.2.

### 3.3. Age-Banded Treatment Thresholds

Framework. The 2021 ESC guideline replaced the older single ≥5%/≥10% mortality cut-offs with three age-banded thresholds for SCORE2 risk (10-year fatal + non-fatal CV events): age < 50 y, high risk ≥ 2.5% and very-high risk ≥ 7.5%; age 50–69 y, high risk ≥ 5% and very-high risk ≥ 10%; age ≥ 70 y (SCORE2-OP), high risk ≥ 7.5% and very-high risk ≥ 15%. The age-banding is biologically defensible: absolute risk rises monotonically with age, so a uniform cut-off would over-treat older and under-treat younger patients. It introduces a stepwise statin and blood-pressure intensification logic that mirrors the LDL-C goal hierarchy of the 2019 ESC/EAS dyslipidemia guideline (LDL-C < 3.0, <2.6, <1.8, and <1.4 mmol/L for low, moderate, high, and very-high risk, respectively, with a ≥50% baseline reduction) [23].

Emerging evidence. Post-guideline validation work in contemporary European primary care cohorts has suggested that SCORE2 may underestimate 10-year event rates in some populations, particularly older adults, which, in practice, shifts a proportion of patients with apparently “borderline” or “high” risk into higher observed risk strata. These findings do not alter the ESC thresholds but indicate that, in very-high-risk settings such as Romania, clinicians should treat SCORE2-derived categories as a minimum estimate and integrate modifiers and imaging more systematically when risk appears borderline.

### 3.4. Modifiers, Qualifiers, and the Borderline Category

Framework. The 2021 framework does not rely on SCORE2 alone. Class I recommendations require the clinician to also consider, in every patient: (i) frailty and life expectancy; (ii) lifetime CVD risk and treatment benefit; and (iii) “risk modifiers”—psychosocial stress, family history of premature CVD, ethnicity, body-mass index, CAC, and frailty [5]. Lp(a) is recommended for measurement “at least once in each adult’s lifetime” (Class IIa, Level B), reiterated by the EAS 2022 consensus [12]. CAC scoring carries Class IIb (Level B) status in patients with intermediate risk to refine therapeutic decisions; carotid plaque assessment has the same status. This is, in practice, a generous borderline category—but its operationalization is non-prescriptive: the guideline does not tell the clinician when the borderline patient must be referred for CAC or Lp(a), only that he or she may be. The result, observable in real Romanian primary-care practice and confirmed by audits in other European countries, is that modifiers are inconsistently applied and the borderline patient is frequently classified by SCORE2 alone [9,24].

Emerging evidence. In real-world practice, audits from several European countries suggest that these modifiers are applied inconsistently and that many borderline patients are classified and treated based on SCORE2 alone. This gap between guideline wording and implementation supports the need for more deterministic modifier panels or checklists in very-high-risk regions, an approach that is at proposal level rather than guideline endorsed.

### 3.5. Treatment Intensification

Framework. The 2021 ESC framework operationalizes the SCORE2 verdict through three parallel therapeutic axes. LDL-C lowering is recommended with class-I statins in high- and very-high-risk patients, ezetimibe add-on when targets are not met, and PCSK9 inhibitors (alirocumab, evolocumab) and inclisiran (a small-interfering RNA against hepatic PCSK9 mRNA) for residual LDL-C above goal despite maximally tolerated statin + ezetimibe [23,25,26]. The 2019 ESC/EAS dyslipidemia targets remain in force [23]. Blood-pressure control follows a Class I target of <140/90 mmHg for almost all adults, intensified to 120–130/70–80 mmHg in patients who tolerate treatment, particularly those at high or very high risk [5]. The ESC explicitly stops recommending routine aspirin for primary prevention and reserves it for patients judged at very high CV risk and low bleeding risk after shared decision making (Class IIb), in line with the ASPREE, ASCEND, and ARRIVE trials [24,27,28].

Emerging evidence. Post-guideline observational and implementation studies suggest that the 2021 ESC framework increases eligibility for preventive lipid-lowering therapy compared with earlier European guidance, while contemporary evidence continues to support stepwise LDL-C lowering with statin intensification, followed by ezetimibe and, in selected very-high-risk patients, PCSK9-based therapy. At the same time, more recent primary-prevention analyses reinforce the restricted role of aspirin and suggest that net benefit is greatest only in carefully selected individuals, particularly when overall atherosclerotic burden is high, and bleeding risk is low; these data support but do not formally redefine current ESC recommendations.

## 4. The ACC/AHA 2018/2019 Framework

The 2019 ACC/AHA primary-prevention guideline and the closely linked 2018 AHA/ACC multisociety cholesterol guideline together form the dominant North American framework for CV risk assessment in apparently healthy adults [6,7]. The framework is structurally different from the ESC’s, and the differences are clinically substantive—not stylistic. They affect who is screened, how borderline patients are arbitrated, and how aggressively LDL-C is lowered.

### 4.1. Pooled Cohort Equations and 10-Year ASCVD Risk

Framework. The cornerstone of the ACC/AHA framework is the PCE, originally introduced in the 2013 ACC/AHA cholesterol guideline and retained in 2018/2019 [6,7,29]. PCE estimates the 10-year risk of atherosclerotic cardiovascular disease (ASCVD)—non-fatal MI, coronary heart disease death, or fatal/non-fatal stroke—in non-Hispanic White and African American adults aged 40–79 years using age, sex, race, total cholesterol, HDL cholesterol, systolic blood pressure (treated or untreated), diabetes status, and smoking [29]. The 2019 revision did not replace PCE but augmented it with explicit risk-enhancing factors and a structured role for CAC scoring.

Emerging evidence. The PREVENT equations, published in 2023–2024 by AHA/ACC/multisociety committees, rederive the model using contemporary multi-ethnic US data, with kidney and metabolic components and a wider age window (30–79 years), but have not yet replaced PCE in formal guideline recommendations [30].

### 4.2. Risk Categories and Decision Thresholds

Framework. PCE outputs are stratified into four categories, each with a defined therapeutic implication [6]: low risk (<5%, lifestyle counselling); borderline (5% to <7.5%, selective statin initiation guided by risk-enhancing factors and shared decision making); intermediate (7.5% to <20%, moderate-intensity statin recommended; CAC is a Class IIa option to reclassify uncertain cases); and high risk (≥20%, high-intensity statin, Class I). The 2018 cholesterol guideline prescribes LDL-C threshold-of-action (≥1.8 mmol/L on therapy) and percent-reduction goals (≥50% LDL-C reduction in high-risk adults) without specifying absolute LDL-C targets—a frequent source of confusion when European clinicians compare the two documents [7,23].

Emerging evidence. Subsequent validation studies have reported that PCE overestimates risk in some contemporary US and external populations, leading clinicians to adopt more conservative statin use when adjusting for perceived overestimation. These data, while informative, have not yet prompted a formal recalibration of the guideline text and should be interpreted as post-guideline evidence rather than as a change in the recommended thresholds.

### 4.3. Risk-Enhancing Factors: A Structured Amplifier Panel

Framework. The decisive operational difference between 2019 ACC/AHA and 2021 ESC is the explicit, named list of risk-enhancing factors that the US guideline requires clinicians to consider in borderline and intermediate patients [6]. These include: family history of premature ASCVD; persistently elevated LDL-C ≥ 4.1 mmol/L (160 mg/dL); chronic kidney disease (eGFR 15–59 mL/min/1.73 m^2^ with or without albuminuria); metabolic syndrome; chronic inflammatory conditions (rheumatoid arthritis, psoriasis, HIV/AIDS); premature menopause (<40 years) and pregnancy-associated conditions including pre-eclampsia; high-risk race/ethnicity (e.g., South Asian); persistent triglycerides ≥ 2.0 mmol/L; high-sensitivity CRP ≥ 2.0 mg/L; Lp(a) ≥ 125 nmol/L (or ≥50 mg/dL); apolipoprotein B ≥ 130 mg/dL; and ankle-brachial index < 0.9. The 2019 guideline names all these prospectively—a deterministic list—and instructs clinicians to use them to upgrade borderline patients to statin therapy and to consider CAC in those remaining uncertain. The 2021 ESC document mentions most of the same modifiers but does not present them as a unified named panel.

Emerging evidence. Observational data suggest that formalizing such amplifier panels into simple checklists improves consistency of statin initiation and may reduce undertreatment in high-risk individuals. However, the specific operational forms of these checklists remain at proposal level and are not codified in ACC/AHA text.

### 4.4. The Structured Role of Coronary Artery Calcium

Framework. CAC scoring carries a Class IIa, Level B-NR recommendation in the 2019 ACC/AHA guideline for borderline and intermediate ASCVD-risk adults in whom the decision to initiate statin therapy is uncertain after consideration of risk-enhancing factors [6,31]. The CAC algorithm is unusually prescriptive for a US document: CAC = 0 in low–intermediate risk patients without other compelling indications justifies withholding a statin (the “power of zero,” supported by 10- and 15-year MESA outcomes) [15,32]; CAC 1–99 is interpreted in context, with statin “favored” in adults ≥ 55 years; and any CAC ≥ 100 Agatston units or ≥75th percentile for age and sex moves the patient to the statin-recommended group regardless of PCE output. The supporting evidence base—including the EISNER randomized trial showing that CAC results change clinician behavior without inducing downstream harm [33] and the SCOT-HEART 5-year outcomes showing a 41% relative reduction in coronary death/MI with CT-guided management [34]—has matured considerably since 2019. From a feasibility standpoint, ROMA-CV deliberately treats coronary artery calcium and carotid/femoral ultrasound as complementary, not interchangeable, gating tools. This selective imaging gate is consistent with contemporary evidence syntheses on preventive imaging, which highlight coronary artery calcium and arterial ultrasound as key tools for refining cardiovascular risk assessment beyond clinical scores. But its deployment in Romania is currently constrained by scanner availability, reimbursement heterogeneity, and concerns about cumulative radiation exposure in younger adults. In contrast, vascular ultrasound is already widely available in Romanian cardiology and vascular laboratories, with no radiation burden, lower unit cost, and more flexible scheduling, making it a pragmatic first-line imaging tool in many regions.

Emerging evidence. Subsequent trials and cohort analyses (e.g., MESA, EISNER, SCOT-HEART) have strengthened the prognostic and behavioral-change value of CAC and coronary CT, without yet triggering an upgrade of CAC recommendations in ACC/AHA classes or levels. Their integration into non-US practice pathways, such as ROMA-CV, therefore, represents a hypothesis-generating extrapolation, aligned with, but not mandated by, current guidelines.

### 4.5. Age Coverage and Population Calibration

Framework. Two further differences are worth highlighting. PCE covers ages 40–79; SCORE2 covers 40–69; SCORE2-OP covers 70–89. Both frameworks share the same lower age boundary of 40 years (Section 6.1). The 2019 ACC/AHA guideline does, however, include explicit recommendations to consider lifetime ASCVD risk in adults aged 20–39 and to act on individual risk-enhancing factors regardless of age, particularly Lp(a) and severe hypercholesterolemia [6,7]. PCEs were derived from US cohorts and are known to overestimate risk in some contemporary US and external populations [35,36]; SCORE2 corrects for this through regional recalibration, at the cost of the country-coefficient problem analyzed in Section 6.2. Neither approach is biologically superior—they are different solutions to the same calibration trade-off.

Emerging evidence. Observational registries published after ESC 2021 document a rise in myocardial infarction incidence in adults under 40 years, highlighting an emerging evidence signal that is not yet fully reflected in current guideline frameworks and reinforcing the need for structured risk assessment in this age group

## 5. Head-to-Head Comparison and Clinical Implications

Table 1 lays out a decision-by-decision comparison of the two frameworks. Four observations are operationally important. First, convergence on therapy: once a patient is correctly classified as high risk, the two frameworks converge on high-intensity statin, an LDL-C reduction of ≥50% (or absolute target < 1.4–1.8 mmol/L), intensive blood-pressure control, and glycemic and weight management. Disagreement is upstream, at patient selection. Second, divergence on borderline arbitration: the ACC/AHA framework names its arbitration tools explicitly (risk-enhancing factors → CAC), whereas the ESC lists modifiers but leaves the sequence of use to clinician judgement [5,6]. In high-mortality regions such as Romania, where SCORE2 already pushes most middle-aged patients toward the high-risk band, the ESC’s permissive framing is comparatively less helpful than the US algorithmic logic. Third, Lp(a) sits in an ambiguous place in both frameworks; both endorse measurement, neither use it as a gating decision. The 2022 EAS consensus and the 2022 AHA scientific statement are clearer than either guideline: Lp(a) should be measured once in every adult’s lifetime, and elevation should trigger more aggressive control of all other modifiable risk factors [12,13]. Fourth, both frameworks have substantially stepped back from routine aspirin use in primary prevention, restricting it to select patients at high CV risk and low bleeding risk (Class IIb) [6,24,27,28].

## 6. Structural Limitations of SCORE2 in a Very-High-Risk Setting

### 6.1. The Age-40 Blind Spot

The 2021 ESC guideline establishes SCORE2 as the first-line risk-estimation tool for apparently healthy adults but defines its operational range as 40 to 69 years, with SCORE2-OP extending to those aged 70 and above [5,20]. Below age 40, the algorithm returns no quantitative output. The guideline offers two surrogates—lifetime risk and risk-age—but designates them as Class IIb tools “to motivate behavioral change” rather than as decision instruments comparable to the 10-year SCORE2 chart. The equivalent ACC/AHA tools begin at 40 (PCE) and 30 (PREVENT) [6,30], leaving the European clinician with the most restrictive lower age limit among major risk calculators. For operational clarity, the present ROMA-CV proposal prioritizes PREVENT as the preferred quantitative tool for adults aged 30–39 years, while retaining JBS3 and polygenic risk scores as secondary or context-dependent adjuncts rather than core algorithmic components.

This omission is not theoretical. Contemporary registries from both sides of the Atlantic document a rise in MI incidence in adults under 40. A Massachusetts General Hospital analysis covering 2000–2016 reported that roughly one in five patients hospitalized with a first MI was 40 years of age or younger, and that the proportion of MI patients in this very-young subgroup increased by approximately 2% per year for ten consecutive years [37]. Subsequent reviews in the European and Balkan literature confirm the trend and emphasize that traditional risk factors—smoking, hyperlipidemia, hypertension, male sex, obesity, and family history of premature cardiovascular disease—together with non-traditional contributors such as substance abuse, thrombophilia, immune disease, and psychosocial stress, dominate the etiological profile in this age group [38,39]. None of these patients can be classified prospectively by SCORE2.

The Progression of Early Subclinical Atherosclerosis (PESA) study examined 4184 asymptomatic employees aged 40–54 years with imaging of six vascular territories and detected subclinical atherosclerosis in 63% of participants; 41% showed extensive disease across multiple territories, and even in those classified as low 10-year risk by the Framingham score, half had imaging-detectable disease [14,40]. Carotid and ilio-femoral ultrasound detected plaque more frequently than CAC at this age—a key practical observation for Romanian protocols relying on widely available vascular ultrasound rather than CT [14]. If two-thirds of asymptomatic adults aged 40–54 already have demonstrable atherosclerosis, the same biological process is necessarily underway—and treatable—in their younger counterparts. A risk calculator that begins precisely at the upper bound of this cohort cannot, by construction, capture the early-onset disease it is meant to predict.

Premature MI under age 40 is etiologically heterogeneous, and many drivers are invisible to the five-variable SCORE2 equation: heterozygous familial hypercholesterolemia, affecting approximately 1 in 250–500 individuals worldwide and undiagnosed in over 90% globally [41,42]; elevated Lp(a), present at risk-raising levels in roughly 20–28% of the adult population [12,43]; spontaneous coronary artery dissection, accounting for ~4% of acute coronary syndromes (ACS) overall and >35% of ACS in women under 60 and 43% of pregnancy-associated MIs [44,45]; substance-use triggers (cocaine elevates relative MI risk 23.7-fold in the hour after use) [46,47]; and inflammatory and autoimmune disease, HIV, adverse pregnancy outcomes, premature menopause, and severe obstructive sleep apnea. A calculator built around five conventional variables cannot, by design, identify any of these mechanisms.

The cost-effectiveness argument cuts the other way when lifetime exposure is considered. A 35-year-old with Lp(a) 200 nmol/L and a family history of MI at 48 will spend three decades accumulating atherosclerotic burden before SCORE2 even begins to estimate his risk. Initiating moderate-intensity lipid lowering at 35—or merely measuring Lp(a), screening for familial hypercholesterolemia, and intensifying lifestyle counseling—is cheaper and more effective than the inevitable downstream percutaneous coronary intervention, dual antiplatelet therapy, and cardiac rehabilitation that follow a 45-year-old’s first event. In the UK Biobank, individuals in the top 20% of polygenic risk who maintained favorable lifestyles had 46% fewer cardiac events than those with unfavorable lifestyles, and high cardiorespiratory fitness reduced the genetic risk of coronary artery disease by 49% [48]. These returns are concentrated in the pre-40s decades.

Several mature instruments fill the SCORE2 vacuum: the Joint British Societies JBS3 lifetime-risk calculator, applicable from age 30 [49]; SCORE2 lifetime tables included as an appendix to the 2021 ESC guideline [5]; AHA PREVENT, which generates 10- and 30-year ASCVD and heart failure estimates from age 30 [30]; CAC scoring at age 30–40 in selected patients, where longitudinal MESA data confirm graded long-term risk [15,50]; and polygenic risk scores, which contribute most where age-driven traditional risk is still low [51]. None of these tools is perfect; collectively, they restore the quantitative decision support that SCORE2 withholds.

### 6.2. The Country-Coefficient Problem

From a guideline standpoint, region-level recalibration is an endorsed solution to heterogeneous European mortality patterns; however, ecological bias considerations and contemporary mortality shifts suggest that applying country-derived multipliers at the individual level may be vulnerable in very high-risk settings such as Romania. This concern is based on post-guideline validation data and epidemiological reasoning and should be viewed as expert interpretation rather than as a formal guideline revision. To make SCORE2 portable across the east–west and north–south European gradients in CVD burden, the investigators applied a region-level recalibration rather than refitting the model in each country. They grouped European nations into four risk regions using WHO age- and sex-standardized CVD mortality rates [20]. Romania entered the very-high-risk region with an age-standardized CVD mortality of 330.5 per 100,000 (WHO data, 2016), and the 2023 Eurostat update places Romania at 787 circulatory-disease deaths per 100,000—second only to Bulgaria (923) and far above the EU average of 313 [1,20,52]. From a public-health perspective, the stratification is defensible: it acknowledges that the background hazard of CVD in Eastern Europe is severalfold higher than in France or Spain. The country coefficient is, by construction, a scalar applied to the baseline hazard that captures the residual, unexplained excess of CV events in each country after accounting for age, sex, smoking, blood pressure, and cholesterol. It is an aggregate descriptor of a population—not a biological property of any single patient.

**Figure 1:**
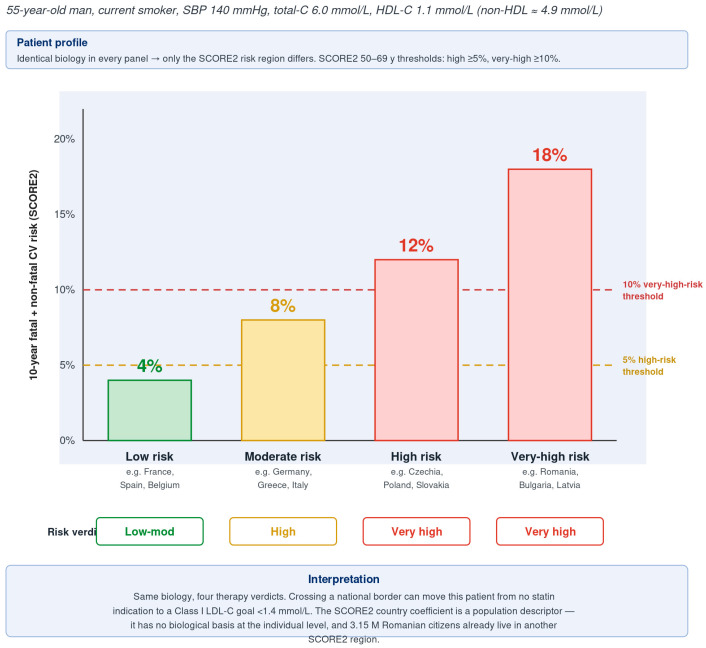
Same patient, four verdicts: the SCORE2 country-coefficient effect. An identical 55-year-old man (current smoker, SBP 140 mmHg, total-C 6.0 mmol/L, HDL-C 1.1 mmol/L) is graded from low–moderate to very-high 10-year CV risk—and from no statin indication to a Class I LDL-C goal <1.4 mmol/L—solely by changing the SCORE2 risk region, with no change in measured biology.

Inferring an individual outcome from a group-level association is the canonical definition of the ecological fallacy in epidemiology [53,54]. Ecological inferences are valid for ecological targets (allocating prevention budgets, planning catheterization-lab capacity, projecting national mortality) but become biased when transferred to the individual scale, because within-group heterogeneity in exposure and outcome is averaged away. Applying a country-derived multiplier to a single patient assumes that this patient’s unmeasured risk profile is identical to the country average. For most individuals, it is not. Consider two Romanian men, both aged 50, both non-smokers, both with systolic blood pressure 130 mmHg and a total-to-HDL cholesterol ratio of 4: SCORE2-very-high places them at roughly the same 10-year risk. Yet one may carry a lifelong Lp(a) of 200 nmol/L and a paternal MI at age 45; the other may have an Lp(a) below 30 nmol/L, no family history, and a fitness level in the top decile-Figure 1. Their true 10-year hazards differ by a factor of two or three [55,56].

The four-region SCORE2 stratification assumes intra-regional homogeneity. Yet within Romania, age-standardized CVD mortality differs by a factor of nearly two between Bucharest-Ilfov and the north-eastern counties, with rural–urban gradients in lipid profiles, hypertension awareness, smoking prevalence, and access to cardiology services that mirror or exceed the differences SCORE2 uses to separate “moderate” from “very high” regions [52]. The European Union guarantees freedom of movement under Articles 20–21 of the Treaty on the Functioning of the European Union, and Romanians have used that right more than any other nationality: Eurostat data for 2023–2024 show 3.15 million Romanian citizens—roughly 16% of the resident population—living in another EU Member State, the highest absolute and relative figure of any Member country [57,58]. A 50-year-old Romanian who relocates to the Netherlands does not, on the day of relocation, undergo a biological transition from a very-high to a low SCORE2 region; conversely, a Dutch retiree who settles in Constanța is not biologically reassigned. Lifelong exposure to diet, pollution, healthcare access, genetics, and behavior travels with the patient; the country chart does not.

Country-level recalibration is only as current as the mortality data feeding it. The published SCORE2 multipliers rest on WHO statistics anchored to the mid-2010s [20]. CVD mortality in Romania has since been disrupted by the COVID-19 pandemic, with documented excess cardiovascular deaths during 2020–2022, and by changes in case fatality after the national STEMI network expansion. None of these dynamics is reflected in the chart a clinician opens today. Empirically, in a contemporary primary-care cohort of 205,548 Dutch adults, the observed 10-year CVD event rate was 10.1% against a SCORE2-predicted 6.2%, with observed-to-expected ratios of 1.54 in women and 1.68 in men, and approximately 35% of patients aged ≥ 50 with predicted risk below 10% experienced actual risks above that threshold—the precise group whom guidelines would withhold from preventive pharmacotherapy [9]. A separate validation in EPIC-Norfolk reported underestimation in men and overestimation in women, with SCORE2-OP performing poorly in older adults even after recalibration [59]. The Cochrane systematic review of CVD risk scores echoes these findings: discrimination is fair, but calibration is inconsistent across settings [60]. To date, we are not aware of published individual-level validation analyses of SCORE2 calibration in Romanian primary-care cohorts; the examples cited are drawn from Dutch and UK populations and are used here to illustrate general calibration behavior rather than to imply that the magnitude of miscalibration in Romania is known [61]. The ecological fallacy argument regarding country-level coefficients should therefore be interpreted as a biologically and epidemiologically plausible concern that requires formal validation in Romanian data, rather than as a proven miscalibration estimate.

Three operational consequences follow. First, SCORE2 output should be treated as a floor, not a ceiling: it estimates the risk of the average Romanian with the patient’s measured traditional risk factors, and the true individual risk is almost always modulated upward or downward by unmeasured determinants. Second, the algorithm must be coupled with an individual modifier panel—Lp(a), family history, polygenic risk, where available, subclinical atherosclerosis imaging—that supersedes the country coefficient whenever individual data are richer than population data. Third, for the mobile EU citizen, clinicians should default to the patient’s lifelong exposure history rather than the current postal code; in practice, this means using the very-high-region chart for a Romanian patient regardless of where he or she currently lives and emphasizing the individual modifier panel for any cross-border patient.

### 6.3. Underused Lp(a) and Subclinical Atherosclerosis Imaging

Lipoprotein(a) is a low-density-lipoprotein-like particle covalently linked to apolipoprotein(a). It is genetically determined, stable across the lifespan after early adulthood, and largely impervious to diet, exercise, statins, or PCSK9 inhibitors [12]. The 2022 EAS consensus statement confirms causality for ASCVD, aortic valve stenosis, ischemic stroke, peripheral artery disease, and—at the highest tail of the distribution—heart failure and CV mortality [12,13]. The association is continuous and log-linear; the pragmatic cut-offs (rule-out < 30 mg/dL or <75 nmol/L; rule-in > 50 mg/dL or >125 nmol/L) exist for operational convenience, not pathobiology.

Roughly one in five adults globally carries Lp(a) above the rule-in threshold; in a 48-country cross-sectional study of 48,135 patients with established ASCVD, 27.9% had Lp(a) > 50 mg/dL, 20.7% > 70 mg/dL, and 12.9% > 90 mg/dL [43]. Younger and female patients have higher levels—exactly the demographic SCORE2 underestimates. In intermediate-risk cohorts, Lp(a) measurement reclassifies approximately 10–15% of patients across treatment thresholds [12]. A 2025 Family Heart Foundation analysis demonstrated that Lp(a) confers a continuous increment in recurrent-event risk with no plateau; patients at ≥300 nmol/L had an approximately 40% higher 5-year event rate than those at <15 nmol/L, with the relationship holding across sex, race and diabetes status [55,56].

Specific Lp(a)-lowering therapies are entering phase-3 outcomes evaluation. Pelacarsen (antisense oligonucleotide; Lp(a)HORIZON, NCT04023552) lowers Lp(a) by approximately 70% in patients with prior ASCVD [62]. Olpasiran (small-interfering RNA; OCEAN(a)-DOSE phase 2 showed >95% reductions sustained for up to a year off-treatment [63]; OCEAN(a)-Outcomes is enrolling). Lepodisiran (siRNA; ACCLAIM-Lp(a), NCT06292013) achieved a 93.9% time-averaged Lp(a) reduction from days 60–180 after a single dose and 88.5% reduction at 360 days in the phase-2 trial [64,65]. Muvalaplin, the only oral candidate, blocks apo(a)–apoB assembly; the 12-week KRAKEN phase-2 trial showed 85.7% reductions at the 240 mg dose with 97% of participants achieving Lp(a) < 125 nmol/L [66,67]. First outcome readouts are expected from 2026 onward. Once any of these agents earns approval, Lp(a) measurement transitions from prognostic curiosity to therapeutic gateway—and a population that has not been measured will not be eligible for therapy.

In 2026, CAC scoring by non-contrast cardiac CT remains the most powerful single reclassification tool in primary prevention. MESA cohort follow-up over a median of 11.1 years demonstrated that every doubling of CAC was associated with a 14% relative increment in ASCVD risk, independent of traditional risk factors, and all participants with CAC > 100 had a >7.5% 10-year risk regardless of age, sex, or race/ethnicity [15]. Conversely, CAC = 0—present in approximately half of asymptomatic middle-aged adults—was associated with 10-year event rates almost uniformly below 5% [32,68]. The 2018 AHA/ACC cholesterol guideline and the 2019 ACC/AHA primary-prevention guideline jointly elevated CAC to Class IIa for shared decision making in adults with borderline or intermediate 10-year ASCVD risk [6,7]. The 2021 ESC guideline mentions CAC only at Class IIb, an asymmetry that is increasingly difficult to defend given the evidence base [5,69]. The SCOT-HEART trial added a complementary signal: in patients with stable chest pain, allocation to coronary CT angiography plus standard care reduced the 5-year composite of coronary heart disease death or non-fatal MI from 3.9% to 2.3% (HR 0.59, 95% CI 0.41–0.84) [34]. The ongoing SCOT-HEART 2 trial extends this question into primary prevention [70].

Where CT capacity is limited, carotid and ilio-femoral B-mode ultrasound provides a robust, radiation-free assessment of plaque burden. PESA showed that ultrasound-detected plaque is more frequent than CAC in the 40–54-year band (60% vs. 18%) and identifies disease earlier in the natural history of atherosclerosis [14]. In Romania, vascular ultrasound capacity is widely available and reimbursed, making this modality the natural primary screening tool for adults under 40 with an amplifier or family history. Additional amplifiers—ankle-brachial index < 0.9, apolipoprotein B ≥ 130 mg/dL, polygenic risk scores in selected centers, hs-CRP ≥ 2 mg/L, chronic inflammatory disease, and sex-specific factors—each independently reclassify CV risk and are detailed in the 2019 ACC/AHA risk-enhancing factor list [6,71,72,73,74,75,76,77,78].

## 7. The ROMA-CV Algorithm: A Proposed Country-Specific Pathway

The argument developed in Section 5 and Section 6 yields a set of constraints that any contemporary Romanian primary-prevention framework should satisfy. To improve interpretability, we explicitly distinguish below between (i) components directly supported by guideline-endorsed recommendations in the ESC 2021 and ACC/AHA 2018/2019 frameworks and (ii) components that reflect post-guideline evidence synthesis or proposal-level operationalization within ROMA-CV.

The ROMA-CV algorithm is built around five explicit design principles. (i) Life-course rather than purely 10-year, because the 10-year horizon of SCORE2 is short relative to the natural history of atherosclerosis [79,80]. (ii) Individual-level rather than region-level inputs; the country coefficient is used only when no individual-level data are available and is superseded whenever modifier information exists. (iii) Stepwise escalation: cheap, universal inputs come first; expensive imaging is selective and triggered only when reclassification would change therapy. (iv) Action-linked, so that every classification maps explicitly to an LDL-C goal, a blood-pressure target, an antiplatelet decision, a lifestyle prescription, and a follow-up interval. (v) Romanian operational reality: the algorithm assumes the actual laboratory, imaging, primary-care, and reimbursement landscape of Romania in 2026 and ties each step to existing or planned infrastructure under the 2025–2030 National Cardiovascular Health Plan [81]. The acronym ROMA-CV stands for Risk Of Multifactorial Atherosclerosis—CardioVascular. To minimize selective evidence assembly, each ROMA-CV step is formally cross-mapped to its underpinning guideline recommendations and principal primary-study domains in Appendix B. Framework-based elements of ROMA-CV include retention of SCORE2/SCORE2-OP as the quantitative starting point for European primary prevention, once-in-a-lifetime lipoprotein(a) measurement as a guideline-supported risk modifier, consideration of risk-enhancing factors/amplifiers, and selective use of coronary artery calcium or carotid/femoral ultrasound to refine uncertainty in non-high-risk patients. By contrast, the deterministic sequencing of these elements within a mandatory Romanian pathway—particularly the use of a structured amplifier checklist, the proposal to treat SCORE2 as a quantitative floor rather than a definitive verdict, and the country-specific integration of imaging when clinical suspicion exceeds calculated risk—should be interpreted as an emerging-evidence-informed proposal rather than as a current ESC- or ACC/AHA-endorsed algorithm. Accordingly, ROMA-CV should be read not as a replacement for existing guidelines, but as a hypothesis-generating implementation model that organizes endorsed recommendations and emerging evidence into a pragmatic sequence for a very-high-risk national setting.

### 7.1. Step 1—Universal Baseline Assessment

Every adult aged ≥ 18 years presenting to a primary care or cardiology clinic—at first contact and at routine periodic health examinations—receives a structured baseline assessment. The history and examination cover personal and family history of premature ASCVD (M < 55 y, F < 65 y), smoking status (pack-years), alcohol and substance use, occupational and shift-work exposure, sleep quality (STOP-BANG), psychosocial stress, and obstetric history (pre-eclampsia, gestational diabetes, premature menopause) [82]. Blood pressure (two seated, standardized readings), body mass index, and waist circumference complete the assessment. The laboratory panel includes fasting total cholesterol, LDL-C, HDL-C, non-HDL-C, triglycerides, apolipoprotein B, where laboratory capacity permits, fasting glucose or HbA1c, serum creatinine with estimated glomerular filtration rate, urinary albumin-to-creatinine ratio, and a complete blood count. Crucially, lipoprotein(a) is measured ONCE in every adult’s lifetime [12], at the patient’s first cardiovascular risk assessment, and the result is flagged in the electronic record so it is not repeated unnecessarily. Within ROMA-CV, an elevated Lp(a) does not trigger experimental therapy but mandates more aggressive control of all modifiable risk factors already endorsed by ESC and ACC/AHA guidelines: earlier and more intensive LDL-C lowering (including high-intensity statins and ezetimibe, and PCSK9-inhibitor consideration in very-high-risk patients), tighter blood-pressure control, strict smoking cessation, and prioritized use of subclinical-atherosclerosis imaging in the presence of borderline quantitative risk. In other words, Lp(a) acts as a deterministic amplifier of existing evidence-based interventions rather than as a gate to presently investigational Lp(a)-targeted agents. Universal baseline assessment and once-in-a-lifetime lipoprotein(a) measurement are fully concordant with existing ESC/EAS and ACC/AHA Class I–IIa recommendations and can be considered guideline aligned.

Lifestyle counseling—Mediterranean-pattern dietary advice, ≥150 min/week moderate aerobic activity plus resistance training, smoking-cessation support, alcohol moderation, and sleep hygiene—is delivered to every patient regardless of subsequent risk score, in accordance with ESC 2021 Class I recommendations [5]. Pelacarsen, olpasiran, and other Lp(a)-lowering agents are therefore explicitly treated as “future options” in ROMA-CV, pending trial readouts and reimbursement decisions, and are not assumed to be routinely available in the current Romanian setting.

### 7.2. Step 2—Quantitative Risk Estimation, Anchored but Acknowledged as a Floor

Quantitative scoring is then applied with age-tiered tools. Age-tiered quantitative risk estimation uses guideline-endorsed SCORE2/SCORE2-OP charts but interprets estimated risk as a minimum (floor) that should be upgraded when modifiers or imaging indicate higher hazard; this interpretive stance is based on emerging validation evidence and is not specified in the current guideline text. For ages 40–69, SCORE2 is used with the very-high-risk region chart, supplemented by SCORE2-Diabetes for type 2 diabetes [22] and, where validated, by SCORE2 for chronic kidney disease. For ages ≥ 70, SCORE2-OP is used with explicit recognition of its known limitations [59]. For ages < 40, SCORE2 is not used as a decision tool; instead, the algorithm calculates a 30-year lifetime risk (SCORE2 lifetime appendix tables [5], JBS3 [49], or AHA PREVENT 30-year output [30]) and reports a risk-age to the patient, which is a more persuasive driver of behavior than an abstract percentage. Critically, every SCORE2 output is interpreted as a floor, not a ceiling, acknowledging the country-coefficient critique developed in Section 6.2 [9,20]. The electronic decision-support template carries a one-line caveat under each SCORE2 result: “This estimate describes the average Romanian patient with these traditional risk factors. Individual risk may be higher or lower depending on amplifiers measured in Step 3.” The 2022 Hungarian comparison of SCORE versus SCORE2 illustrates the extent of numerical migration the new chart already produces—97.7% of men aged 40–50 were classified as low–moderate by SCORE versus 32.4% by SCORE2—and reinforces the need for an additional layer of individualization beyond the score itself [83].

For adults aged 30–39 years, in whom SCORE2 does not provide a routine quantitative estimate, ROMA-CV uses the AHA PREVENT equations (30-year risk framework) as the preferred quantitative complement to clinical judgment, because PREVENT is specifically derived for adults aged 30–79 years and allows earlier capture of long-horizon cardiovascular risk. In this age stratum, severe hypercholesterolemia, markedly elevated lipoprotein(a), family history of premature ASCVD, diabetes, chronic kidney disease, or major inflammatory disease should be treated as automatic amplifiers that prompt intensified preventive management irrespective of low short-term absolute risk. Polygenic risk scores may be considered exploratory adjuncts where locally available, but they are not required for routine implementation of ROMA-CV and are not positioned as first-line decision tools in the present version of the algorithm.

### 7.3. Step 3—The Mandatory Amplifier Checklist

The single most important procedural innovation of ROMA-CV is the conversion of the ESC 2021 list of Class-IIb modifiers into a mandatory checklist. Every Step-1 baseline is followed by an explicit review of: Lp(a) ≥ 50 mg/dL (≥125 nmol/L); family history of premature ASCVD (M < 55, F < 65); chronic kidney disease stage ≥ 3 or persistent urinary albumin-to-creatinine ratio ≥ 30 mg/g; persistent LDL-C ≥ 4.1 mmol/L (≥160 mg/dL) or triglycerides ≥ 2.0 mmol/L despite lifestyle measures; ankle-brachial index < 0.9 in patients with metabolic syndrome, diabetes or smokers ≥ 50 y [72]; hs-CRP ≥ 2 mg/L combined with at least one additional traditional risk factor [75,76]; chronic inflammatory disease (rheumatoid arthritis, psoriasis, systemic lupus erythematosus, inflammatory bowel disease, HIV) [77]; adverse pregnancy outcomes, premature menopause, polycystic ovary syndrome [78]; severe obstructive sleep apneas, shift work, high environmental-pollution exposure; and a high-risk polygenic score where available [73]. The presence of any single amplifier triggers a one-tier upward classification of the SCORE2 output (low–moderate → high; high → very high, and so on). This procedural rigor matches the operational specificity of the ACC/AHA risk-enhancing factor list [6] and removes the discretion that, in practice, becomes omission. The mandatory amplifier checklist converts guideline-listed modifiers into a deterministic panel; this operationalization goes beyond ESC wording and should be regarded as a hypothesis-generating proposal grounded in ACC/AHA “risk-enhancing” logic.

### 7.4. Step 4—Selective Subclinical-Atherosclerosis Imaging as the Arbiter

Imaging is invoked only when reclassification would change therapy—that is, in the borderline and intermediate bands, in any amplifier-driven uncertainty, and in patients hesitant about lifelong pharmacotherapy. For adults aged 40–75 years with a 10-year SCORE2 of 5–20% or a strong family history of premature ASCVD, CAC scoring by non-contrast cardiac CT is the preferred arbiter. CAC = 0 supports deferring statin (unless diabetes, current smoker, familial hypercholesterolemia, or markedly elevated Lp(a) are present), with recheck CAC in 5–10 years [68]; CAC 1–99 indicates moderate risk, lifestyle intensification, and consideration of moderate-intensity statin; CAC ≥ 100 indicates high risk and statin initiation regardless of SCORE2 output [15]. For adults aged < 40 with one or more amplifiers, and for any-age patients at centers without CT, carotid and femoral ultrasound is preferred: plaque ≥ 1.5 mm in any territory reclassifies the patient as high risk and triggers therapy intensification [84], while the absence of plaque defers pharmacotherapy unless familial hypercholesterolemia, very high Lp(a), or strong family history dictates otherwise. The choice between CAC and ultrasound is dictated by patient age, availability, and family history strength, in accordance with PESA’s observation that ultrasound outperforms CAC for early plaque detection in adults under 55 [14]. Selective CAC or carotid/femoral ultrasound arbitration is supported by ESC and ACC/AHA Class IIa/IIb recommendations for imaging in borderline/intermediate risk, but the specific thresholds and decision rules used in ROMA-CV are at proposal level and not yet guideline endorsed.

### 7.5. Therapeutic Mapping

Each final risk category leads to an explicit set of actions (Table 2), harmonized with ESC 2021 (prevention), ESC 2024 (chronic coronary syndromes [85]), and EAS 2022 [5,23,85].

**Figure 2:**
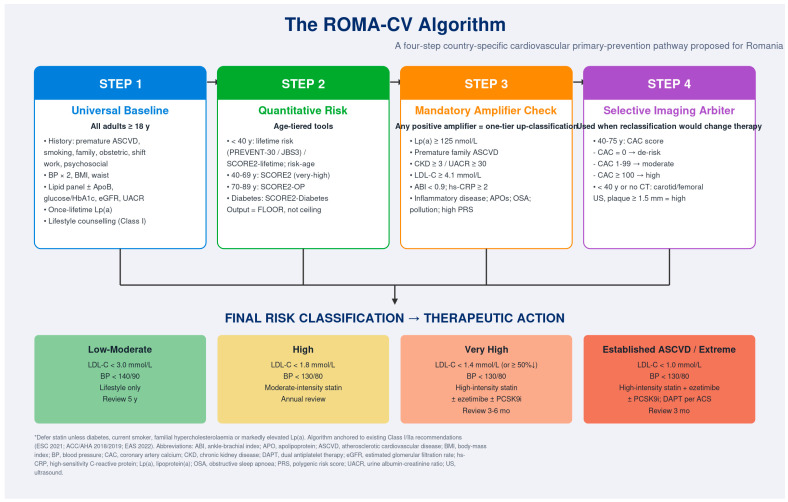
Schematic of the proposed four-step ROMA-CV algorithm. Step 1: universal baseline assessment with once-in-a-lifetime Lp(a). Step 2: age-tiered quantitative risk estimation (SCORE2/SCORE2-OP for 40–89 y; lifetime risk or PREVENT 30-year for <40 y), treated as a floor not a ceiling. Step 3: mandatory amplifier checklist (any positive amplifier triggers one-tier up-classification). Step 4: selective imaging arbitration with CAC (40–75 y) or carotid/femoral ultrasound (<40 y or no CT). Outputs map deterministically to LDL-C, blood-pressure, antithrombotic, and follow-up decisions (Table 2).

Schematic representation of the four step ROMA-CV algorithm is describe in Figure 2.

## 8. Implementation Roadmap for Romania

ROMA-CV is intentionally designed to be deployable, not aspirational. Three operational realities frame the roadmap. First, the 2025–2030 National Plan for the Prevention and Control of Non-Communicable Diseases commits approximately €204 million and the establishment of 10–12 regional prevention centers, providing the physical infrastructure and reimbursement vehicle for a national risk-stratification program [17]. Second, the EU Cardiovascular Health Plan, formally released by the European Commission on 16 December 2025, names prevention, early detection and screening, and treatment and care as the three pillars of EU-wide CV policy through 2030, and explicitly invites Member States to align national plans with these pillars [86,87]. Third, Romania already operates a functioning national stroke registry (RES-Q), 26 contributing hospitals, and a Romanian Society of Cardiology infrastructure capable of supporting national audit programs [88,89,90]. Implementation requires sequencing rather than invention.

### 8.1. Phase 1 (Months 0–12): Governance and Laboratory Readiness

The most important Phase 1 deliverable is administrative: a single national protocol for cardiovascular primary prevention, jointly ratified by the Romanian Society of Cardiology, the Ministry of Health, and the National Health Insurance House, which adopts ROMA-CV as the reference pathway for the regional prevention centers. The protocol should specify (i) the four-step decision sequence, (ii) the reimbursement codes for Lp(a), high-sensitivity CRP, urinary albumin-to-creatinine ratio, lipid profile including ApoB and CAC scoring, and (iii) the data-capture variables required for national audit. Three laboratory dependencies must be solved: Lp(a) assay harmonization (WHO/IFCC SRM-2B traceability, results in nmol/L preferred and mg/dL secondary) [12]; reimbursement of ApoB alongside non-HDL-cholesterol [12,71]; and inclusion of hs-CRP and urinary albumin-to-creatinine ratio as default analytes in the prevention-center baseline panel [6,91].

### 8.2. Phase 2 (Months 12–30): Regional Rollout and Clinician Training

The 10–12 regional prevention centers form the operational backbone. We propose a hub-and-spoke architecture in which each center is staffed by at least one preventive cardiologist, one clinical biochemist, and one allied health professional (dietitian or nurse case manager), and is supported by primary-care referrals through a single secure electronic pathway. Each center should be equipped with, or have access to, a 16-slice or greater multidetector CT scanner capable of CAC scoring [92] on a defined referral timeline. The Hungarian SCORE-to-SCORE2 reclassification analysis demonstrated that 60–65% of middle-aged men shifted from “low/moderate” to “high” risk on switching algorithms; primary-care clinicians who do not understand why are unlikely to act on the new classification [83]. A short, standardized continuing-medical-education module (recognized by the Romanian College of Physicians) covering SCORE2 mechanics, Lp(a) interpretation, the amplifier checklist, and CAC referral indications should be a precondition of accreditation as a referring physician to the regional center [83,89].

### 8.3. Phase 3 (Months 30–60): Integration, Audit, and Outcomes

Phase 3 converts the operational system into a national auditable program. Three components are essential: integration of ROMA-CV outputs with the existing electronic health record infrastructure and the prescribing system used for reimbursement of statins, ezetimibe, PCSK9 inhibitors and inclisiran; a national audit program modelled on the RES-Q stroke registry [88], with quarterly dashboards and annual public reporting on documented SCORE2 (or lifetime risk under age 40), once-lifetime Lp(a) measurement, documented amplifier checklist for borderline patients, LDL-C target attainment, and three outcome metrics (incident MI, incident ischemic stroke and all-cause CV mortality), stratified by region and socioeconomic decile [4,8]; and evaluation of population-level uptake of cardiac rehabilitation and the cardiovascular polypill (SECURE demonstrated that a single polypill containing aspirin, ramipril, and atorvastatin reduces CV death by 33% and the four-component MACE endpoint by 24% in post-MI patients, with Polish, Hungarian, and Czech sites and external generalizability to Romania) [93].

### 8.4. Phase 4 (Months 60+): Prospective Cohort and Recalibration

The most strategically important Phase 4 component is the prospective follow-up of the under-40 cohort and of the borderline cohort recategorized by CAC scoring. Without a Romanian cohort, the country will continue to rely on national-level recalibration of foreign-derived data; with a Romanian cohort, the country can, in due course, publish a domestically derived risk model and recalibrate SCORE2 to local biology rather than to a multi-decade-old WHO mortality coefficient. Phase 4 should also include a formal economic evaluation; modeling work from other very-high-risk European settings suggests that Lp(a)-targeted screening is cost-effective at conventional thresholds when paired with intensive risk-factor management in carriers [94]. A Romanian-specific evaluation would inform whether to extend Lp(a) measurement from once a lifetime in adults to cascade screening of first-degree relatives—a step endorsed by NICE in the UK but not yet by either ESC or ACC/AHA [12,13,95].

### 8.5. Equity and Cross-Border Considerations

Two cross-cutting issues must be addressed throughout Phases 1–4. The first is equity: the PURE study showed that the gradient of preventable CVD by socioeconomic status is steeper in middle-income than in high-income countries and persists even when risk-factor profiles are favorable [96]. A national risk-assessment program concentrated in urban centers will widen rather than narrow the gradient. The roadmap, therefore, requires explicit budget commitment to mobile prevention units, telecardiology-supported screening in rural primary care, and Romanian-language patient-facing materials calibrated to a low-literacy reading level. The second is the European single-market reality: approximately 3.15 million Romanian citizens live in other EU Member States, and many oscillate between countries for work and care [16,86]. A pragmatic mitigation is mutual recognition of the once-a-lifetime Lp(a) measurement, the amplifier checklist, and the CAC result across EU healthcare systems, supported by EU-level data-portability frameworks.

### 8.6. Quantitative Targets for 2030

Defined narrowly, success in 2030 would be: (i) ≥80% of adults aged 40–69 in Romania with a documented ROMA-CV verdict in their primary-care record; (ii) ≥70% of Romanian adults with at least one lifetime Lp(a) measurement; (iii) ≥60% of statin-eligible high-risk patients achieving the ESC/EAS LDL-C target; and (iv) a measurable absolute decline in the standardized circulatory-disease mortality rate from 787 per 100,000 in 2023 toward the EU 2030 indicative target of <500 per 100,000 [1,86].

## 9. Limitations

Methodological transparency is further supported by Appendix A (search and selection framework) and Appendix B (evidence mapping of each ROMA-CV step to its guideline Class/Level and key studies). This article is a narrative review and therefore carries the inherent limitations of the genre: although the search strategy was structured and the inclusion criteria were prespecified, no formal risk-of-bias assessment was performed for individual studies, no quantitative synthesis was attempted, and the selection of supporting evidence necessarily reflects the authors’ judgment. The SANRA framework was applied to maximize transparency, but the design does not approximate a systematic review.

The ROMA-CV algorithm is a proposal. It has not been prospectively validated in any cohort, Romanian or otherwise, and no claim is made about its discrimination, calibration, or net reclassification index relative to SCORE2 alone. Every individual algorithm step is supported by Class I or Class IIa recommendations in ESC 2021 or ACC/AHA 2018/2019, or by Level A/B evidence from primary sources, but the composite four-step pathway has not been tested. Until a prospective Romanian cohort or—ideally—a pragmatic cluster-randomized trial comparing ROMA-CV against SCORE2 alone is completed, ROMA-CV should be regarded as hypothesis-generating and as a framework for ongoing methodological refinement rather than a substitute for the current standard of care.

Three additional caveats deserve explicit acknowledgment. First, the country-coefficient critique applies equally, in principle, to any region-calibrated risk model and is not unique to SCORE2; the practical case for individualization made here would extend to PCE in the United States or QRISK in the United Kingdom. Second, the imaging recommendations depend on infrastructure that is unevenly distributed across Romanian counties; the implementation roadmap addresses this through the planned regional prevention centers, but uneven uptake will produce uneven outcomes and must be measured. Third, the algorithm assumes accurate ascertainment of family history, sex-specific risk factors, and lifestyle exposures in routine primary-care practice; data-quality audits will be essential.

In addition, no internal proof-of-concept simulation or decision-analytic model was performed to estimate how many Romanian adults would be reclassified by ROMA-CV compared with SCORE2 alone, or whether such reclassification would translate into improved treatment allocation. Accordingly, the present manuscript should be interpreted as a mechanistically grounded and guideline-based proposal rather than a quantitatively validated reclassification model. A key limitation is that the manuscript does not present Romanian cost-effectiveness modelling for universal once-in-a-lifetime Lp(a) measurement, and that actionable Lp(a)-lowering therapies (such as pelacarsen and olpasiran) remain investigational at the time of writing. ROMA-CV therefore constrains the clinical consequence of elevated Lp(a) to intensification of guideline-mandated risk-factor control and imaging, rather than assuming immediate access to specific Lp(a)-targeted drugs. Formal economic evaluations in Romanian primary-prevention cohorts will be required to quantify the value of routine Lp(a) testing and to incorporate future Lp(a)-lowering therapies into the pathway once efficacy, safety, and reimbursement profiles are established.

A pragmatic constraint is that CAC scoring, although evidence-based, is not yet uniformly accessible or reimbursed across Romanian regions, and CT capacity is unevenly distributed between tertiary centers and peripheral hospitals. ROMA-CV therefore explicitly allows carotid/femoral ultrasound to serve as the primary subclinical-atherosclerosis gate in settings where CAC is unavailable or not reimbursed, reserving CAC for centers with established CT workflows and clear local funding. The algorithm should be revisited as the National Plan’s imaging investments mature, including any future earmarking of CT capacity specifically for preventive CAC assessment.

Finally, the article is written from a Romanian vantage. The same critique we have applied to SCORE2 in Romania could productively be applied by colleagues in Bulgaria, Hungary, Latvia, Lithuania, Slovakia, and other very-high-risk EU regions, all of whom share the same structural mismatch between a population-calibrated score and an individually variable biological exposure. A cluster of country-specific operational algorithms, sharing the SCORE2 backbone but adding regionally appropriate amplifier handling, would be the natural maturation of European primary-prevention practice.

## 10. Future Directions

Four lines of work follow directly from the proposal. First, a prospective Romanian validation cohort built on the SEPHAR IV blood-pressure cohort [97] and the EUROASPIRE Romanian arm [98], with at least 10 years of follow-up, is the indispensable next step. Such a cohort can deliver a domestically derived recalibration of SCORE2, an empirical estimate of ROMA-CV reclassification yield, and the evidence base for a national audit program. Second, a pragmatic cluster-randomized trial comparing primary-care clinics implementing ROMA-CV against control clinics implementing SCORE2 alone—with primary outcomes of statin uptake, LDL-C target attainment, and time to incident CV event—is methodologically feasible within the National Plan’s timeframe. Third, the algorithm should be tested in adolescents and young adults (ages 15–39) through cascade family screening triggered by Lp(a) and familial hypercholesterolemia findings, integrated with school-based and occupational health programs. Fourth, the algorithm’s portability should be explored in collaboration with cardiology societies in other very-high-risk EU Member States, with the aim of harmonizing the amplifier checklist while retaining country-specific quantitative anchors. A priority methodological aim for Romania is the generation of individual-level SCORE2 and ROMA-CV validation data in primary-care and community cohorts (even at the level of a single regional registry), including calibration plots by age and sex and assessment of reclassification between country-coefficient-based risk and observed event rates. Such data would provide a more balanced test of the country-coefficient hypothesis than extrapolation from Dutch or EPIC-Norfolk cohorts, and would allow the ecological-fallacy concern to be either confirmed or refuted in the Romanian setting.

Methodologically, future iterations should test machine learning approaches to integrate SCORE2, amplifier presence, and imaging results into a single calibrated probability, while retaining the deterministic transparency that primary-care clinicians require. The integration of polygenic risk scores will likely become more clinically relevant as costs fall and as the under-40 cohort moves into the SCORE2 window. Finally, once Lp(a)-lowering agents earn approval, the algorithm’s gating role for therapy eligibility should be reassessed. A priority next step is an internal proof-of-concept modeling exercise in a representative Romanian primary-prevention cohort, comparing SCORE2 alone with ROMA-CV for (i) the proportion of patients reclassified upward or downward, (ii) the change in statin eligibility and LDL-C target assignment, and (iii) decision-analytic measures such as clinically interpretable reclassification tables and, where appropriate, category-based net reclassification indices.

## 11. Conclusions

The 2021 ESC and 2018/2019 ACC/AHA primary-prevention frameworks have transformed cardiovascular risk assessment into a quantitatively defined population-level decision rule [5,6,7]. Their convergence on treatment intensity once a patient is correctly classified as high risk is one of the more impressive achievements of modern guideline-based medicine [5,7,23]. Their divergence is upstream—at the point of patient selection—and that divergence has clinical and public-health consequences that scale with the absolute mortality burden of the population being served. In Romania, with a standardized circulatory disease mortality rate of 787 per 100,000 in 2023 (second only to Bulgaria within the EU) and approximately 3.15 million citizens residing in other Member States, those upstream design choices matter more than in almost any other European setting [1,16].

This narrative review has identified four structurally significant shortcomings of the SCORE2-based ESC framework as it functions in a very-high-risk country: (i) an age floor of 40 years that excludes the population in which premature MI is most preventable [10,11]; (ii) a country-level calibration coefficient applied at the individual level, whose biological basis evaporates as soon as the patient crosses an EU internal border; (iii) a Class-IIa endorsement of Lp(a) measurement honored more in the breach than the observance [12,13]; and (iv) a Class-IIb endorsement of CAC imaging that, given the SCOT-HEART and MESA outcomes’ evidence, is arguably underemphasized relative to its prognostic information yield [15,32,34,99]. None of these is a flaw in the mathematics of SCORE2; each is a design choice that can be operationally corrected within the existing evidence base.

The ROMA-CV algorithm proposed here is the operational consequence of those corrections. It retains SCORE2 as the quantitative spine in the 40–69-year age range, adds explicit lifetime-risk handling in the <40 cohort, makes Lp(a) measurement and a structured amplifier checklist mandatory rather than discretionary, and assigns CAC and carotid/femoral ultrasound an explicit arbitration role for borderline patients. Every step is supported by a Class I or Class IIa recommendation in either the ESC 2021 or ACC/AHA 2018/2019 framework, or by Level A/B evidence from a peer-reviewed primary source. The algorithm is designed to be implemented through Romania’s 2025–2030 National Cardiovascular Plan and its 10–12 regional prevention centers and is aligned with the EU Cardiovascular Health Plan [17,86,87]. ROMA-CV is explicitly framed as a proposal: external validation in a Romanian cohort, ideally complemented by a pragmatic cluster-randomized implementation trial, is the next required step before adoption in routine practice. The mathematics, the evidence, and the financing already exist; the limiting reagent is operational standardization.

## Figures and Tables

**Table 1:** Head-to-head comparison of the 2021 ESC and 2018/2019 ACC/AHA primary-prevention frameworks. Columns summarize guideline-endorsed constructs; footnotes indicate where contemporary validation or emerging data diverge from guideline assumptions.

Decision Node	ESC 2021 (Europe)	ACC/AHA 2018/2019 (USA)
Primary risk score	SCORE2/SCORE2-OP/SCORE2-Diabetes	Pooled Cohort Equations (PCE); PREVENT under transition
Age window covered	40–69 (SCORE2); 70–89 (SCORE2-OP)	40–79 (PCE); 30–79 (PREVENT)
Adults < 40 years	Lifetime risk/risk-age (Class IIb)	Lifetime ASCVD risk considered; action on Lp(a), FH
Calibration approach	Four European risk regions (country-level)	US-derived (see footnote b)
Outcome predicted	Fatal + non-fatal CV events (MI, stroke)	Atherosclerotic CVD (MI, CHD death, stroke)
Risk categories	Low/moderate/high/very-high (age-banded)	Low/borderline/intermediate/high
Borderline arbitration	Permissive modifier consideration	Named risk-enhancing factor panel
Lp(a) measurement	Once-lifetime (Class IIa, Level B)	Risk-enhancing factor ≥ 125 nmol/L
CAC scoring	Class IIb (Level B)	Class IIa, Level B-NR
LDL-C target framing	Numeric absolute targets per risk stratum	Threshold + ≥50% relative reduction
Statin intensity (high risk)	High-intensity ± ezetimibe ± PCSK9i	High-intensity ± ezetimibe ± PCSK9i
Blood-pressure target	<140/90 (Class I); 120–130/70–80 (intensified)	<130/80 (Class I)
Aspirin in primary prevention	Class IIb, restricted	Class IIb, restricted
Polypill	Not formally recommended	2025 update considers in selected patients
Validation in modern cohorts	Post-guideline validation: Underestimation in ~35% of Dutch primary care (see footnote a).	Post-guideline validation: Overestimation in modern US data (see footnote b).

(a) Post-guideline validation data from Dutch primary-care cohorts indicate that SCORE2 underestimates 10-year cardiovascular risk in a proportion of patients, particularly in lower socioeconomic and selected ethnic subgroups. (b) Post-guideline validation data from contemporary US and external cohorts indicate that the PCE framework tends to overestimate 10-year ASCVD risk, contributing to more conservative statin use in some settings. Abbreviations: ASCVD, atherosclerotic cardiovascular disease; CAC, coronary artery calcium; CHD, coronary heart disease; CV, cardiovascular; ESC, European Society of Cardiology; FH, familial hypercholesterolemia; LDL-C, low-density-lipoprotein cholesterol; Lp(a), lipoprotein(a); MI, myocardial infarction; PCE, Pooled Cohort Equations; PCSK9i, proprotein convertase subtilisin/kexin type 9 inhibitor; SCORE2, Systematic COronary Risk Evaluation 2; SCORE2-OP, SCORE2-Older Persons.

**Table 2:** ROMA-CV therapeutic mapping by final risk category.

Final Risk	LDL-C Goal	BP Target	Pharmacotherapy	Follow-Up
Low–moderate	<3.0 mmol/L	<140/90	None; lifestyle only	5 y
High	<1.8 mmol/L	<130/80	Moderate-intensity statin	Annual
Very high	<1.4 mmol/L (or ≥50% reduction)	<130/80	High-intensity statin ± ezetimibe ± PCSK9i	3–6 months
Established ASCVD/extreme	<1.0 mmol/L	<130/80	High-intensity statin + ezetimibe ± PCSK9i; DAPT per ACS guideline	3 months

Abbreviations: ACS, acute coronary syndrome; ASCVD, atherosclerotic cardiovascular disease; BP, blood pressure; DAPT, dual antiplatelet therapy; LDL-C, low-density-lipoprotein cholesterol; PCSK9i, proprotein convertase subtilisin/kexin type 9 inhibitor.

## Data Availability

No new data were created or analyzed in this study. All data discussed are available in the cited primary sources. The annotated search strategy and the cross-mapping to article sections are available from the corresponding author upon reasonable request.

## Supplementary Materials

The following supporting information can be downloaded at: https://www.mdpi.com/article/10.3390/jcm15145490/s1, Supplementary Table S1. SANRA Adherence Checklist for This Narrative Review.

## Abbreviations

ABI, ankle-brachial index; ACC, American College of Cardiology; ACS, acute coronary syndrome; AHA, American Heart Association; ApoB, apolipoprotein B; ASCVD, atherosclerotic cardiovascular disease; BMI, body-mass index; BP, blood pressure; CAC, coronary artery calcium; CCTA, coronary computed-tomography angiography; CHD, coronary heart disease; CKD, chronic kidney disease; CT, computed tomography; CV, cardiovascular; CVD, cardiovascular disease; DAPT, dual antiplatelet therapy; EAS, European Atherosclerosis Society; eGFR, estimated glomerular filtration rate; ESC, European Society of Cardiology; EU, European Union; FH, familial hypercholesterolemia; HDL-C, high-density-lipoprotein cholesterol; HR, hazard ratio; hs-CRP, high-sensitivity C-reactive protein; IFCC, International Federation of Clinical Chemistry; LDL-C, low-density-lipoprotein cholesterol; Lp(a), lipoprotein(a); MACE, major adverse cardiovascular events; MESA, Multi-Ethnic Study of Atherosclerosis; MI, myocardial infarction; PCE, Pooled Cohort Equations; PCSK9i, proprotein convertase subtilisin/kexin type 9 inhibitor; PESA, Progression of Early Subclinical Atherosclerosis; PRS, polygenic risk score; ROMA-CV, Risk Of Multifactorial Atherosclerosis—CardioVascular; SANRA, Scale for the Assessment of Narrative Review Articles; SCAD, spontaneous coronary artery dissection; SCORE2, Systematic COronary Risk Evaluation 2; SCORE2-OP, SCORE2-Older Persons; SGLT2, sodium-glucose co-transporter 2; siRNA, small interfering RNA; STEMI, ST-elevation myocardial infarction; UACR, urinary albumin-to-creatinine ratio; WHO, World Health Organization.

## Appendix A. Search Strategy and Study Selection Framework

This appendix is intended to improve methodological transparency for the narrative review *Beyond SCORE2: Rethinking Cardiovascular Risk Assessment in a Very-High-Risk European Setting—A Narrative Review and Proposal of the ROMA-CV Algorithm for Romania*. The main manuscript already states that the review was conducted as a structured narrative review in accordance with SANRA, searched MEDLINE (PubMed), Embase, and the Cochrane Library through 30 April 2026, and supplemented database searches with targeted website searches, ClinicalTrials.gov, and reference-list screening. The present appendix expands on those methods to provide a reproducible search framework and a transparent article-selection workflow, including representative operational search terms used to implement the concept blocks described in the main Methods section.

### Appendix A.1. Review Scope

The review addressed three linked questions: (1) how the 2021 ESC SCORE2/SCORE2-OP framework and the 2018/2019 ACC/AHA Pooled Cohort Equations framework compare in primary cardiovascular prevention; (2) which structural limitations of SCORE2 are likely to be clinically important in Romania and similar very-high-risk European settings; and (3) which evidence-supported modifications could be assembled into the proposed ROMA-CV conceptual framework.

### Appendix A.2. Databases and Supplementary Sources

The literature search covered MEDLINE via PubMed, Embase, and the Cochrane Library from database inception to 30 April 2026. Supplementary searches were performed on the websites of the European Society of Cardiology, American College of Cardiology, American Heart Association, European Atherosclerosis Society, National Lipid Association, the National Institute for Public Health of Romania, the European Commission, and the Romanian Ministry of Health. ClinicalTrials.gov was searched for late-phase trials of lipoprotein(a)-lowering and inflammation-targeting agents, and Eurostat and the World Health Organization databases were searched for epidemiologic and population-level context. Reference lists of retrieved guidelines, consensus statements, and major reviews were also screened for additional eligible sources.

### Appendix A.3. Core Search Concepts

The manuscript reports that the core PubMed strategy combined four concept blocks: primary cardiovascular prevention/risk estimation, guideline frameworks, risk modifiers, and the Romanian or Eastern European implementation context. Those blocks were operationalized as follows.

#### Appendix A.3.1. Concept Block 1: Primary Prevention and Risk Estimation

- “SCORE2”
- “SCORE2-OP”
- “SCORE2-Diabetes”
- “Pooled Cohort Equations”
- “PREVENT equations”
- “cardiovascular risk assessment”
- “primary cardiovascular prevention”
- “ASCVD risk”

#### Appendix A.3.2. Concept Block 2: Guideline Frameworks

- “ESC guideline”
- “2021 ESC prevention guideline”
- “ACC/AHA guideline”
- “2019 ACC/AHA primary prevention guideline”
- “2018 cholesterol guideline”
- “primary prevention guideline”

#### Appendix A.3.3. Concept Block 3: Risk Modifiers and Reclassification Tools

- “lipoprotein(a)” OR “Lp(a)”
- “coronary artery calcium” OR “CAC”
- “carotid plaque”
- “femoral plaque”
- “subclinical atherosclerosis”
- “risk-enhancing factors”
- “family history”
- “polygenic risk”
- “apolipoprotein B”
- “ankle-brachial index”
- “high-sensitivity C-reactive protein” OR “hs-CRP”

#### Appendix A.3.4. Concept Block 4: Implementation Context

- “Romania”
- “Romanian”
- “Eastern Europe”
- “very high risk region”
- “cardiovascular mortality Europe”
- “national cardiovascular plan”

### Appendix A.4. Example Database Strategy

The main manuscript gives the structure of the PubMed search but not the full string. A reproducible example, aligned with the reported search logic, is shown below and can be cited as the representative reconstruction of the search strategy used to identify potentially relevant literature, rather than a prospectively archived line-by-line export.

#### Appendix A.4.1. Example PubMed Search String

(
   “SCORE2”[tiab] OR “SCORE2-OP”[tiab] OR “SCORE2-Diabetes”[tiab] OR
   “Pooled Cohort Equations”[tiab] OR “PREVENT equations”[tiab] OR
   “cardiovascular risk assessment”[tiab] OR “cardiovascular risk assessment”[Mesh] OR
   “primary cardiovascular prevention”[tiab] OR “ASCVD risk”[tiab]
  )
  AND
  (
   “ESC”[tiab] OR “European Society of Cardiology”[tiab] OR
   “ACC/AHA”[tiab] OR “American College of Cardiology”[tiab] OR
   “American Heart Association”[tiab] OR “primary prevention guideline”[tiab] OR
   “cholesterol guideline”[tiab]
  )
  AND
  (
   “lipoprotein(a)”[tiab] OR “Lp(a)”[tiab] OR
   “coronary artery calcium”[tiab] OR “CAC”[tiab] OR
   “carotid plaque”[tiab] OR “femoral plaque”[tiab] OR
   “subclinical atherosclerosis”[tiab] OR “risk-enhancing factors”[tiab] OR
   “apolipoprotein B”[tiab] OR “ankle-brachial index”[tiab] OR
   “high-sensitivity C-reactive protein”[tiab] OR “hs-CRP”[tiab]
  )
  AND
  (
   “Romania”[tiab] OR “Romanian”[tiab] OR “Eastern Europe”[tiab] OR
   “very high risk region”[tiab]
  )

#### Appendix A.4.2. Adaptation to Other Sources

Equivalent concepts were adapted to Embase and the Cochrane Library using database-specific indexing and syntax. Website searches were narrower and document-targeted, prioritizing guideline statements, consensus documents, national policy texts, and official epidemiologic reports relevant to Romania and the European high-risk setting.

### Appendix A.5. Eligibility Criteria

The manuscript prespecified six inclusion categories: current ESC, ACC/AHA, EAS, and NLA prevention or lipid-management guidelines; derivation and validation studies of SCORE2, SCORE2-OP, SCORE2-Diabetes, and the Pooled Cohort Equations; prospective cohorts and randomized trials informing risk modifiers and imaging; consensus statements on lipoprotein(a), inflammation, and lifetime risk; late-phase lipoprotein(a)-lowering trials; and Romanian national surveillance, mortality, or policy documents. Exclusion criteria were narrative reviews without primary data, conference abstracts without subsequent full publication, and articles unavailable in English or Romanian. When several publications described the same dataset, the most recent comprehensive report was paired, where appropriate, with the original derivation paper.

### Appendix A.6. Study-Selection Workflow

Because this was a structured narrative review rather than a PRISMA-registered systematic review, the search was designed to be comprehensive and purposive, not exhaustively tabulated in a systematic-review format. To improve transparency, the following staged workflow can be added to the manuscript or Supplementary Material:

Database and website searches identified records relevant to prevention guidelines, risk calculators, modifiers, imaging, Romanian epidemiology, and implementation policy.

Duplicate or clearly overlapping records were removed at the title level when multiple versions of the same document were retrieved.

Titles and abstracts were screened for direct relevance to at least one of the three review questions.

Full texts were assessed against the prespecified inclusion and exclusion criteria reported in the Methods section.

Included sources were then mapped to one or more synthesis domains: guideline comparison, SCORE2 limitations, lipoprotein(a), imaging/reclassification, implementation in Romania, or algorithm construction.

### Appendix A.7. Suggested Transparency Statement for the Methods Section

Database and supplementary searches yielded 468 records; after removal of duplicates and clearly overlapping records, 321 titles/abstracts and 104 full texts were screened, and 87 sources were included in the final narrative synthesis.

### Appendix A.8. Cross-Mapping of Evidence to Manuscript Sections

**Evidence Domain**	**Main Sections Informed**
ESC and ACC/AHA guideline frameworks	Section 3, Section 4 and Section 5
SCORE2 derivation, calibration, and validation	Section 3 and Section 6
Age-related blind spots and lifetime risk	Section 6.1 and Section 7.2
Lipoprotein(a), imaging, and risk amplifiers	Section 5, Section 6.3, Section 7.3 and Section 7.4
Romanian epidemiology and policy context	Section 1, Section 6.2 and Section 8
Algorithm development and implementation	Section 2.4, Section 7 and Section 8

## Appendix B. Evidence Base and Guideline Support for the ROMA-CV Algorithm

This appendix is provided to reduce the risk of selection bias in the narrative synthesis underpinning ROMA-CV by explicitly mapping each algorithm step to its supporting guideline recommendations and primary-study evidence, restricted to the prespecified inclusion categories reported in the Methods section and Appendix A.

**ROMA-CV Step/Component**	**Evidence Base Supporting the Step**	**Guideline Class/Level or Analogous Evidence Level**
Step 1. Universal baseline assessment, including once-lifetime lipoprotein(a)	This step is supported by the 2021 ESC prevention guideline framework for structured baseline cardiovascular risk assessment and by the recommendation that lipoprotein(a) be measured at least once in each adult lifetime. The manuscript also cites supporting consensus documents and epidemiologic evidence establishing lipoprotein(a) as a prevalent, genetically determined causal risk factor that is not adequately captured by conventional short-term risk scores.	ESC 2021: baseline risk assessment embedded in Class I prevention workflow; lipoprotein(a) measurement Class IIa, Level B. [file:1] (file:1—Available online: https://academic.oup.com/eurheartj/article/42/34/3227/6358713#supplementary-data (accessed on 10 May 2026))
Step 2. Quantitative risk estimation using SCORE2/SCORE2-OP as the core risk spine, interpreted as a floor rather than a ceiling	This step is anchored to the derivation and validation literature for SCORE2 and SCORE2-OP and to the 2021 ESC guideline recommendation that these tools be used as first-line estimators in apparently healthy adults. The manuscript further supports the step by reviewing calibration limitations, age-floor constraints, and complementary lifetime-risk approaches that justify interpreting the numeric score within a broader clinical framework rather than as a self-sufficient endpoint.	ESC 2021: SCORE2/SCORE2-OP used as the primary quantitative framework, Class I, Level B/A as reflected in the guideline-based prevention pathway and derivation-validation evidence. [file:1]
Step 3. Mandatory amplifier checklist to individualize risk beyond the country coefficient	This step is supported by the explicit ACC/AHA risk-enhancing factor framework and by the ESC recommendation to consider modifiers such as family history, psychosocial burden, frailty, coronary artery calcium, and other patient-level factors. In the manuscript, this step is additionally supported by evidence related to lipoprotein(a), chronic inflammatory conditions, family history, lifetime exposure, and other determinants that are biologically individualized but underrepresented in standard calculators.	ACC/AHA 2019: use of risk-enhancing factors in borderline/intermediate prevention decisions, Class IIa, Level B-NR; ESC 2021: modifier consideration integrated into preventive decision-making, generally permissive and less prescriptive, including Class IIa or IIb domains depending on the modifier. [file:1]
Step 4. Selective subclinical-atherosclerosis imaging with coronary artery calcium and/or carotid-femoral ultrasound in uncertain or reclassifiable cases	This step is grounded in the ACC/AHA framework giving coronary artery calcium a structured role in reclassification and in the ESC guideline permitting coronary artery calcium or carotid plaque assessment in selected patients. The manuscript also cites cohort and trial evidence, including PESA, MESA, EISNER, SCOT-HEART, CONFIRM, and related imaging literature, to support imaging-based refinement when traditional risk estimation and clinical modifiers leave uncertainty.	ACC/AHA 2019: coronary artery calcium for decision refinement, Class IIa, Level B-NR; ESC 2021: coronary artery calcium and carotid plaque assessment, Class IIb, Level B. [file:1]
Therapeutic mapping after reclassification: LDL-C lowering, blood-pressure control, and selective aspirin restriction according to the final risk category	The downstream management step is supported by the 2019 ESC/EAS dyslipidaemia guideline, the 2021 ESC prevention guideline, and the 2018/2019 ACC/AHA cholesterol and primary-prevention guidelines. The manuscript explicitly states that no novel treatment thresholds were created and that all action nodes were constrained to existing Class I or Class IIa recommendations or to peer-reviewed Level A/B evidence from major randomized trials and validation studies.	Lipid-lowering and blood-pressure interventions are predominantly supported by Class I guideline recommendations with Level A or B evidence; aspirin in primary prevention is restricted and framed as Class IIb in selected very-high-risk, low-bleeding-risk settings. [file:1]
