# Supplementary material for: Beyond SCORE2: Rethinking Cardiovascular Risk Assessment in a Very-High-Risk European Setting—A Narrative Review and Proposal of the ROMA-CV Algorithm for Romania
Source: J Clin Med. 2026 Jul 13;15(14):5490. doi: 10.3390/jcm15145490 (PMC13412616; doi:10.3390/jcm15145490)
Supplement: Supplementary file 1 [file jcm-15-05490-s001.zip › jcm-4388583-supplementary.pdf]

Supplementary Table S1. SANRA Adherence Checklist for This Narrative Review

Title of manuscript: *Beyond SCORE2: Rethinking Cardiovascular Risk Assessment in a Very-High-Risk European Setting — A Narrative Review and Proposal of the ROMA-CV Algorithm for Romania*

This supplementary table documents how the present narrative review fulfills the six SANRA domains and is intended to support methodological transparency and editorial verification.

SANRA item	Ready-to-paste wording for the supplementary table	Location in manuscript
1. Justification of the article's importance for the readership	The review addresses a clinically important and regionally relevant problem: persistent excess cardiovascular mortality in Romania and the potential mismatch between current risk-estimation frameworks and prevention needs in a very-high-risk European setting. The manuscript explains why reappraisal of SCORE2 is relevant for readers involved in primary prevention, cardiovascular risk stratification, and guideline implementation.	Section 1. Introduction; Abstract
2. Statement of concrete aims or formulation of questions	The manuscript states three explicit aims: (i) to compare the 2021 ESC and 2018/2019 ACC/AHA primary-prevention frameworks, (ii) to examine structural limitations of SCORE2 that are clinically consequential in Romanian practice, and (iii) to propose the hypothesis-generating ROMA-CV algorithm as a country-specific, evidence-based pathway requiring prospective validation.	Final paragraph of Section 1. Introduction
3. Description of the literature search	The review provides a structured description of information sources, databases, search period, supplementary manual searches, core search concepts, eligibility criteria, and synthesis logic. Specifically, MEDLINE/PubMed, Embase, and the Cochrane Library were searched through 30 April 2026, supplemented by guideline society websites, ClinicalTrials.gov, Eurostat, WHO sources, and reference-list screening. Inclusion and exclusion criteria, as well as the rationale for algorithm development, are described in the Methods section.	Sections 2.2–2.4. Materials and Methods
4. Referencing	Referencing is comprehensive and appropriate to the scope of a structured narrative review. The manuscript cites contemporary ESC and ACC/AHA guidelines, derivation and validation studies for SCORE2 and PCE, key cohort studies, imaging studies, lipoprotein(a) and inflammation-related consensus documents, major therapeutic trials, and Romanian/EU epidemiological and policy sources.	Throughout manuscript; especially Sections 1–7 and References

5. Scientific reasoning	The manuscript uses explicit scientific reasoning by moving from epidemiological context and guideline comparison to identification of four structural limitations of SCORE2, then linking each limitation to evidence-based mitigations incorporated into the ROMA-CV proposal. The argument is developed stepwise and constrained to recommendations, evidence levels, and interventions considered deployable in current Romanian practice.	Sections 3–7, especially Sections 5, 6, and 7
6. Appropriate presentation of data	Data and arguments are presented in a structured and reader-oriented format using clearly separated sections, comparative tables, and an algorithmic figure/graphical abstract. The manuscript integrates narrative synthesis with tabular comparison of guideline frameworks and schematic presentation of the proposed ROMA-CV pathway to improve clarity and applicability.	Abstract; Sections 3–7; Table 1; algorithm figure/graphical abstract
